# Supplementary material for: Plocabulin Displays Strong Cytotoxic Activity in a Personalized Colon Cancer Patient-Derived 3D Organoid Assay
Source: Mar Drugs. 2019 Nov 19;17(11):648. doi: 10.3390/md17110648 (PMC6891270; doi:10.3390/md17110648)
Supplement: Supplementary file 1 [file marinedrugs-17-00648-s001.pdf]

marine drugs

Supplementary Materials:

Plocabulin displays strong cytotoxic activity in a personalized colon cancer patient-derived 3D organoid assay

Alba Costales-Carrera[1,2], Asunción Fernández-Barral[1,2], Pilar Bustamante-Madrid[1,2], Laura Guerra[3], Ramón Cantero[3], Antonio Barbáchano[1,2] and Alberto Muñoz[1,2] *

[1] Department of Cancer Biology, Instituto de Investigaciones Biomédicas "Alberto Sols" (CSIC-UAM) and University Hospital La Paz Institute for Health Research (IdiPAZ), Madrid, Spain; amunoz@iib.uam.es

[2] Biomedical Research Networking Centres-Oncology (CIBERONC), Madrid, Spain; abarbachano@iib.uam.es

[3] General surgery and Pathology services University Hospital La Paz, Madrid, Spain; laura.guerra@salud.madrid.org

* Correspondence: amunoz@iib.uam.es; Tel: +34-91-5854451

Figure S1

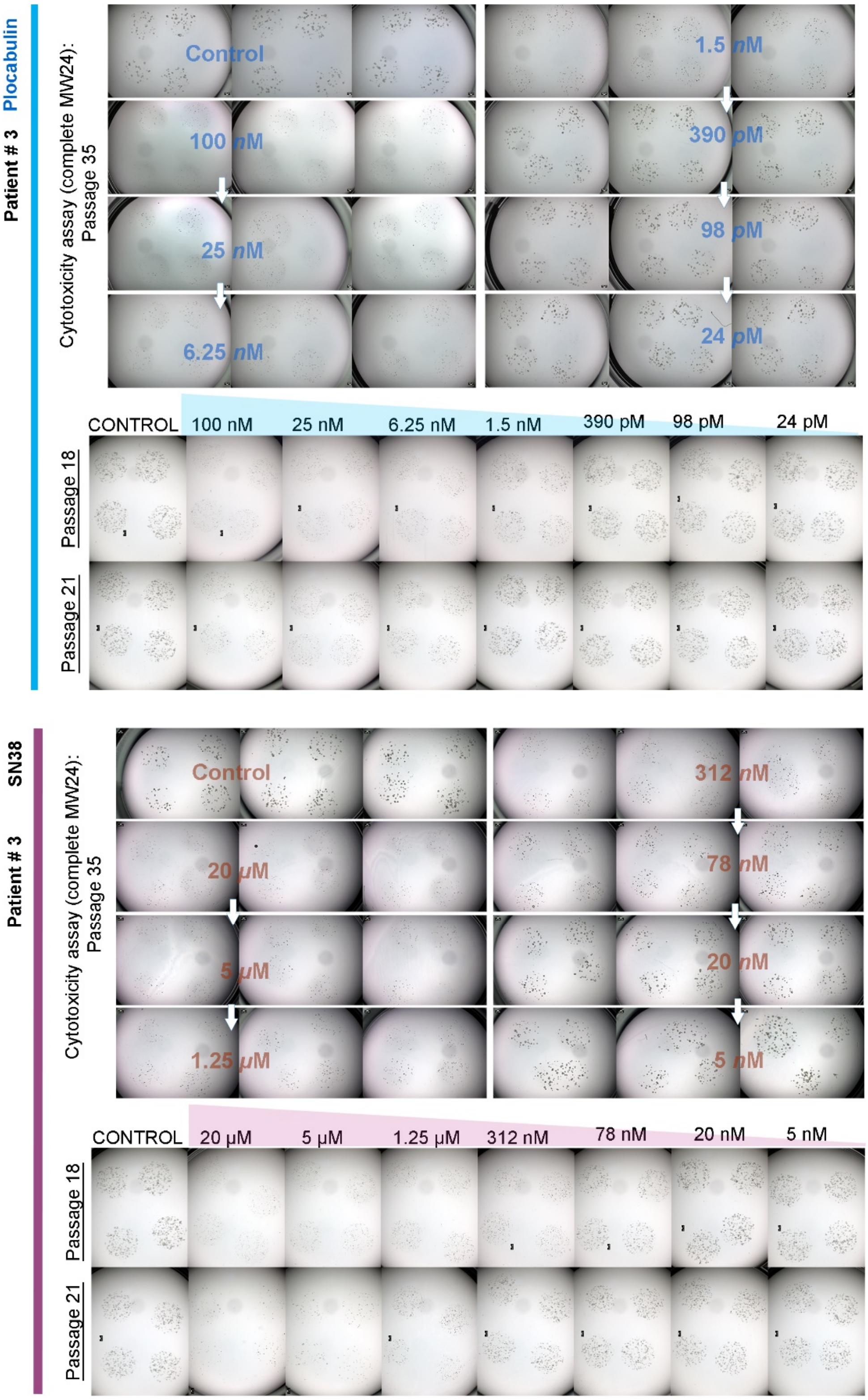

Figure S1. Plocabulin and SN38 action on patient #3 colon tumor organoid 3D culture. Images of assays on passage 35 are mounted resembling the design of a complete 24-well cytotoxicity assay (4-drop triplicates for each dose). For passage 18 and 21 assays, a single representative image per drug dose is shown. Microplate wells were photographed after 4 days of drug treatment using a Leica DFC550 digital camera mounted on a Leica S6D stereomicroscope.

Figure S2

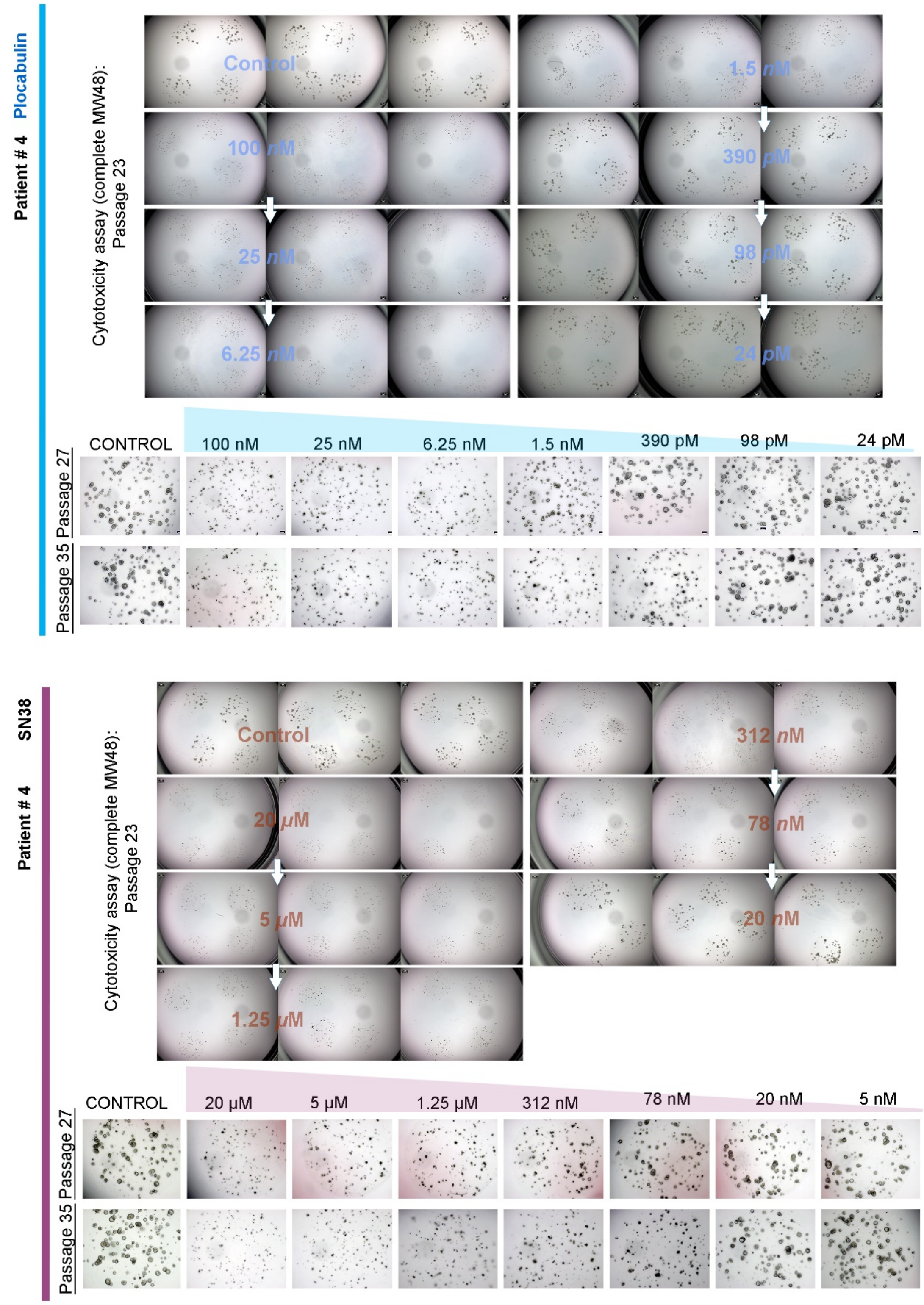

Figure S2. Plocabulin and SN38 action on patient #4 colon tumor organoid 3D culture. Images of assays on passage 23 are mounted resembling the design of a complete 24-well cytotoxicity assay (4-drop triplicates for each dose) and were taken with a Leica DFC550 digital camera mounted in a Leica S6D stereomicroscope. For passage 35 and 27 assays, a representative phase-contrast image of a single drop is shown for each drug dose; images were captured with a Leica DFC550 digital camera mounted on an inverted Nikon TS100 microscope. Microplate wells were photographed after 4 days of drug treatment.

Figure S3

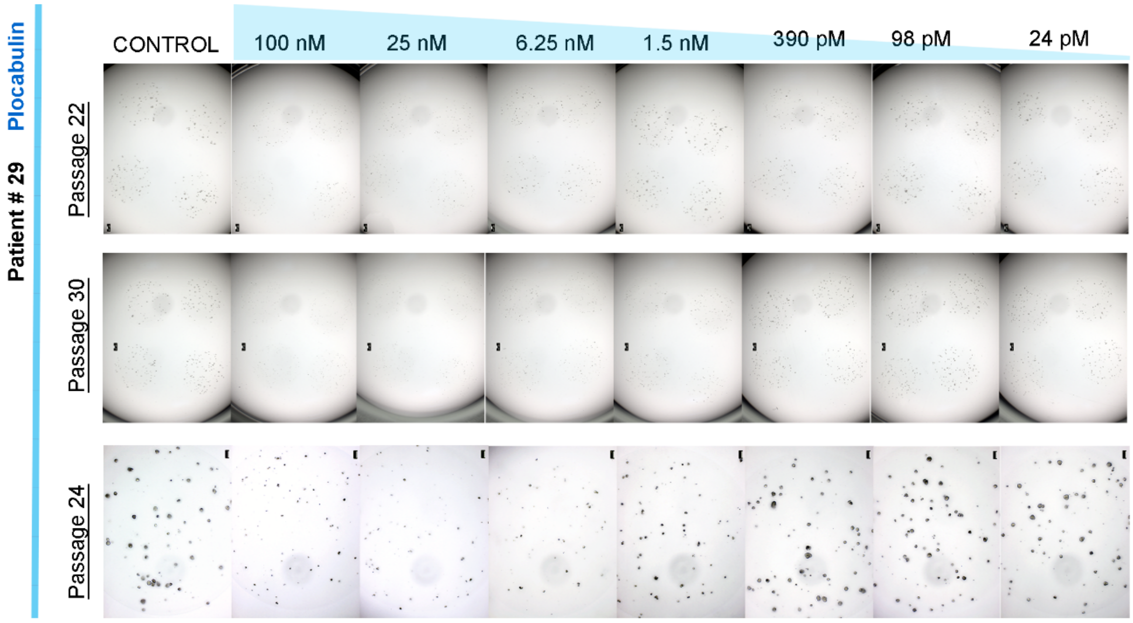

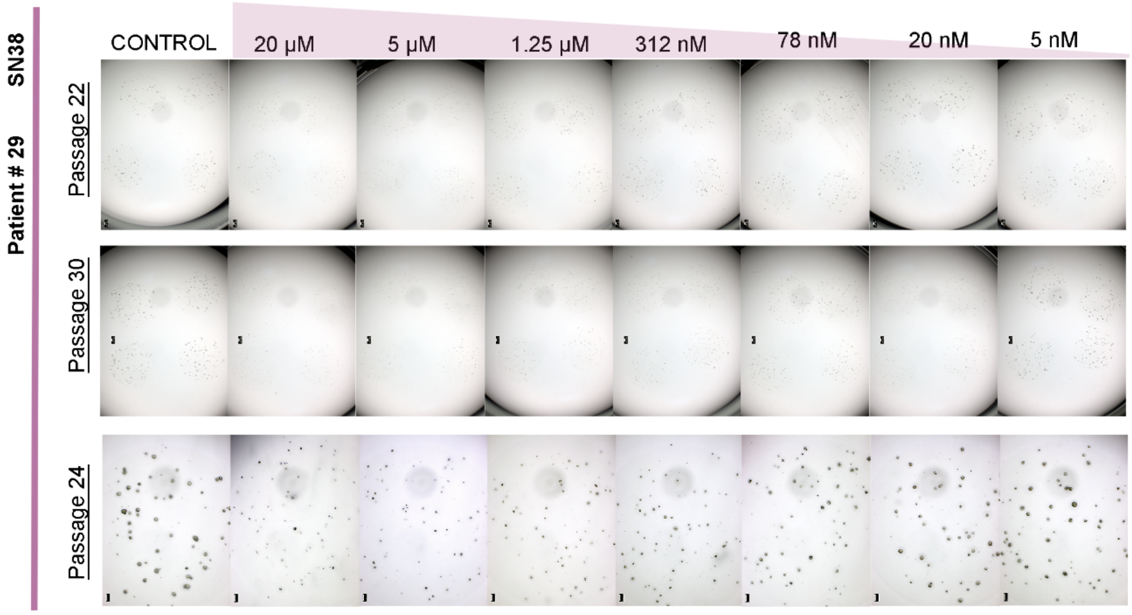

Figure S3. Plocabulin and SN38 action on patient #29 colon tumor organoid 3D culture. For passage 22 and 30 assays, a single representative images per drug dose (4 drops) is shown. Images were taken with a Leica DFC550 digital camera mounted in a Leica S6D stereomicroscope. For assays in passage 24, a phase-contrast image of a single drop is shown for each drug dose; images were captured with a Leica DFC550 digital camera mounted on an inverted Nikon TS100 microscope. Microplate wells were photographed after 4 days of drug treatment.

Figure S4

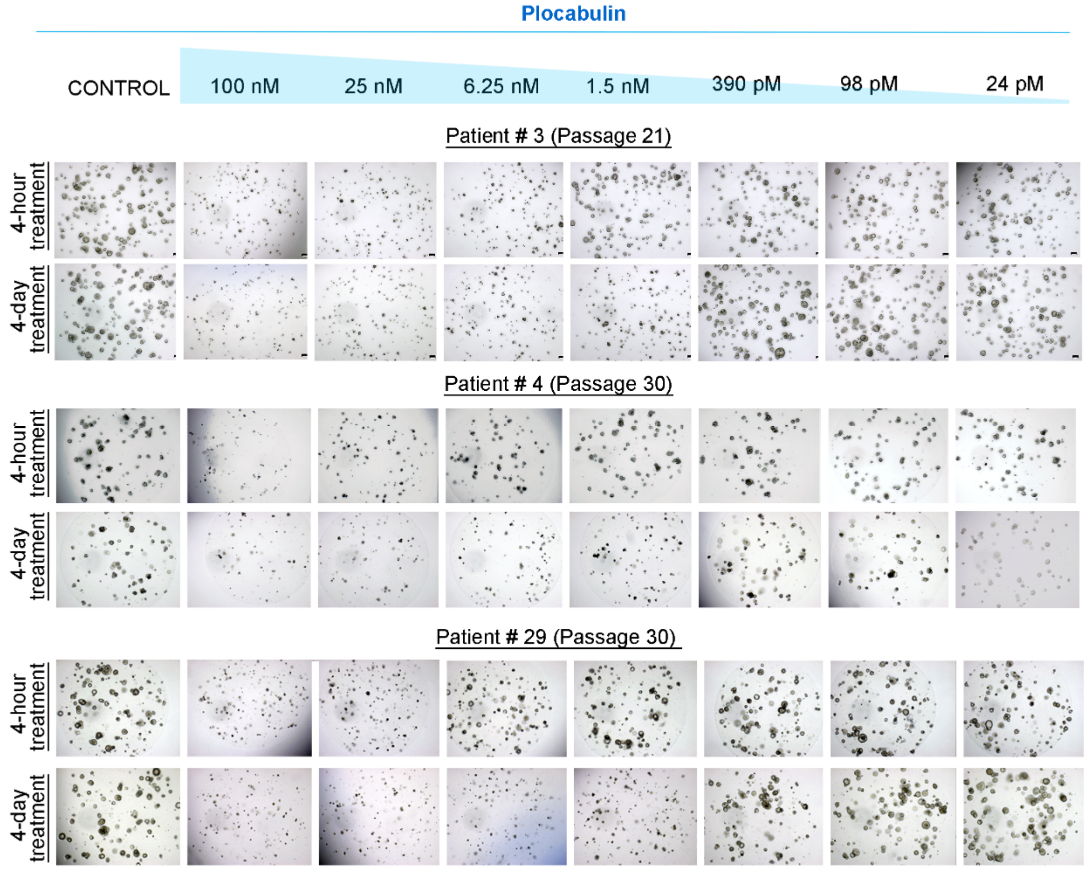

Figure S4. Plocabulin wash-out assays on patient #3, #4 and #29 colon tumor organoids. Organoids were treated with increasing doses of plocabulin that were either removed (and organoids washed) after 4 hours or left for 4 days until the end of the experiment.

Table S1. Quality control parameters of optimization and cytotoxicity assays. In optimization assays, the highest Z-score and the lowest error among control replicates are indicated in **bold**. For all cytotoxicity assays *Z-scores* > 0.5 (excellent).

Organoid size (um)	Nº cells/well	Mean Dose Max	Stdev Dose Max	Mean Control	Stdev Control	Z' score	Replicates Error (%)	Drug
Patient # 3 Optimization assay								
0-20	500	413283	47125	1394000	248765	0.09	16.85	Plocabulin
0-20	1000	658148	69484	4474618	776624	0.33	9.68	Plocabulin
20-40	125	1768308	350356	22015141	800499	0.83	8.87	Plocabulin
**20-40**	**250**	**2746802**	**298942**	**24807637**	**971804**	**0.83**	**6.09**	Plocabulin
40-70	25	350656	40357	6200457	1947150	-0.02	28.28	Plocabulin
40-70	50	870587	267613	16224438	2019466	0.55	14.03	Plocabulin
40-70	100	2105740	289822	22080887	2697896	0.55	9.31	Plocabulin
40-70	200	2756345	597499	24105200	1070690	0.77	12.87	Plocabulin
Patient # 4 Optimization assay								
0-20	500	5154	2708	21285	12923	-1.91	59.51	Plocabulin
0-20	1000	3844	2602	127006	58913	-0.50	45.51	Plocabulin
20-40	125	29795	19670	501911	237200	-0.63	35.98	Plocabulin
**20-40**	**250**	**168246**	**15972**	**1896479**	**83535**	**0.83**	**9.23**	Plocabulin
40-70	25	229719	61523	4300094	859736	0.32	19.86	Plocabulin
40-70	50	57265	39804	1541867	708407	-0.51	47.03	Plocabulin
40-70	100	202549	40242	4140153	1132057	0.11	21.20	Plocabulin
40-70	200	168592	56636	3178922	847947	0.10	26.24	Plocabulin
Patient # 3 Cytotoxic assays								
20-40	250	215145	19580	44358481	2914011	0.80	6.57	SN38
20-40	250	625412	35629	52476972	1340186	0.92	2.55	SN38
20-40	250	974119	53102	42265760	1323605	0.90	3.13	SN38
20-40	250	618436	86867	35739738	1908494	0.83	5.34	Plocabulin
20-40	250	262475	14650	27349327	1444995	0.84	5.28	Plocabulin
20-40	250	1804668	90823	33778793	578087	0.94	1.71	Plocabulin
20-40	250	1014079	151515	44830910	2623823	0.81	5.85	Plocabulin (4-hours)
Patient # 4 Cytotoxic assays								
20-40	250	1355139	176286	21247096	2813298	0.55	13.24	SN38
20-40	250	640569	103270	7561784	920643	0.56	12.17	SN38
20-40	250	1112398	53085	26658938	910771	0.89	3.42	SN38
20-40	250	772899	46941	35435702	2877947	0.75	8.12	Plocabulin
20-40	250	372839	39144	4699088	230572	0.81	4.91	Plocabulin
20-40	250	481200	68757	4480864	502943	0.57	11.22	Plocabulin
20-40	250	3801358	961696	58475645	6284173	0.60	10.75	Plocabulin (4-hours)
Patient # 29 Cytotoxic assays								

20-40	250	2096986	148234	31057958	298495	0.95	0.96	SN38
20-40	250	151329	15999	7538308	646543	0.73	8.58	SN38
20-40	250	219808	22178	6787216	355806	0.83	5.24	SN38
20-40	250	427430	2398	8178260	948348	0.63	11.60	Plocabulin
20-40	250	15606	2393	8808391	225671	0.92	2.56	Plocabulin
20-40	250	1305073	108784	31177552	1165901	0.87	3.74	Plocabulin
20-40	250	458905	45358	30122351	2243235	0.77	7.45	Plocabulin (4-hours)
